# 5.K. Workshop: National and European studies on health literacy in children and adolescents

**DOI:** 10.1093/eurpub/ckac129.317

**Published:** 2022-10-25

**Authors:** Organised by: EUPHA Working Group on Health Literacy, EUPHA-HP, EUPHA-CAPH, Technical University Munich, Robert Koch Institute, Chair persons: Orkan Okan (EUPHA-HP), Kimberley Foley (UK)

## Abstract

Health literacy describes how people use health information to make informed decisions in context of healthcare, disease prevention and health promotion. Enhancing health literacy of populations is critical and in particular important at an early age, which is understood to be more sustainable because health literacy contributes to improved personal health and development. Low health literacy in child and adolescent populations has been linked to worse health outcomes and health disparities in Europe, making health literacy of children and adolescents an important public health topic. Developing and delivering target group specific interventions and services requires precise data generation on the state of health literacy in early age. In the past decade, several conceptual approaches have been undertaken but methodological sound, validated and reliable measurement tools are still scarce. Available systematic reviews show that most tools originated from North America and/or English speaking countries. However, in the past years European researchers have been involved with research on health literacy in childhood and adolescence, the result of which culminated into heavy progress regarding the development of health literacy measurement instruments, including generic health literacy, digital health and mental health literacy. These new developments lead to the availability of validated tools for school-aged children, including primary and secondary school children. The purpose of this workshop is to bring together five contemporary health literacy studies conducted in child and adolescent populations across Europe, including national and European-wide studies. Both methodological findings regarding the measurement tool and empirical data will be introduced. The first presentation emerges from the HLCA HL-Kids project, which has been conducted in Germany and focusses on primary schoolchildren, using the HLS-Child-Q15 tool. The second presentation originates from the Netherlands where the HLS-Child-Q15 tool was adapted to Dutch children. The third presentation aims at health literacy of adolescents in secondary school-age in Germany, by using the MOHLAA-Q tool. The fourth presentation originates from the WHO-led HBSC study, which has been conducted by using the HLSAC questionnaire. The final presentation will focus on the first digital health literacy measurement tool for secondary schoolchildren, which has been developed within a German study in schools. Each project will be given ten minutes to present their findings, including questions, which will be followed by Q&A and an open discussion with the audiences. This workshop offers a forum for researchers, practitioners and policy-makers interested in health literacy measurement in children and adolescents. By dialogue and two-way communication, vivid interaction will be ensured, allow building synergies, and facilitate networking and capacity building.

**Key messages:**

• Health literacy contributes to improved personal health and development of children and adolescents.

• The measurement of health literacy is key to public health intervention success and needs theory driven, validated and reliable instruments.

